# Intrapopulation Metabolic Variation Reflects Growth Differences: A Cross‐Sectional Study on Gammarides

**DOI:** 10.1002/ece3.73544

**Published:** 2026-04-29

**Authors:** Federica De Castro, Ludovico Lezzi, Milad Shokri, Laura Del Coco, Francesco Paolo Fanizzi, Alberto Basset

**Affiliations:** ^1^ Department of Biological and Environmental Sciences and Technologies (DiSTeBA) University of Salento Lecce Italy; ^2^ National Biodiversity Future Centre (NBFC) Palermo Italy; ^3^ LifeWatch ERIC Service Centre Lecce Italy

**Keywords:** cohort, environmental metabolomics, *Gammarus insensibilis*, intrapopulation variability, metabolism, NMR spectroscopy, pace of life

## Abstract

1. Intrapopulation variability in growth trajectories among conspecifics provides a reservoir of phenotypic diversity that can buffer populations against environmental change. From a life‐history and metabolic‐scaling perspective, this variability reflects alternative resource‐allocation strategies—ranging from rapid biomass accretion to energy conservation—that may represent bet‐hedging under fluctuating environments. Biochemical metabolic processes may underpin these diverse strategies, though the mechanisms involved remain poorly understood.
2. A cohort of *Gammarus insensibilis* , a broadly distributed aquatic macroinvertebrate commonly found in transitional water ecosystems, was reared under laboratory conditions for 75 days. Despite being of the same age, individuals displayed marked size differences and were subsequently classified by body size into Small (S), Medium (M), and Large (L) groups as proxies for slow, intermediate, and fast growers.
3. To test the occurrence of a biochemical basis of this intrapopulation variability, a single‐organism ^1^H NMR metabolomics approach was applied to *Gammarus insensibilis* individuals.
4. Metabolic profiling of each animal revealed discrete “fingerprints” rather than a uniform baseline state, with at least two major metabolic pathways significantly different between S and L size individuals. These pathways included alanine, aspartate, and glutamate as well as arginine metabolism. Medium‐sized individuals displayed intermediate profiles with unique metabolite ratios pointing to phenotypic plasticity.
5. These results highlight the utility of ^1^H NMR spectroscopy in resolving individual metabolic states and identifying anabolic pathways linked to growth phenotypes, suggesting that phenotype‐dependent performance under fluctuating conditions may maintain intrapopulation variability.

## Graphical abstract

For the first time in *Gammarus insensibilis* , a single‐organism ^1^H NMR metabolomics approach was applied to investigate the intrapopulation variability in growth trajectories showing how divergent size classes, despite being of the same age, reflect alternative resource‐allocation strategies.

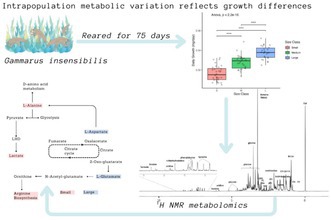

## 1 Introduction

A notable characteristic across diverse benthic invertebrate populations, and within many other biological groups, is the pronounced variability in individual growth rates observed among conspecifics (Burton et al. 2011; Goodrich and Clark 2023; Shokri et al. 2019; Stark et al. 2025). This intrapopulation variability, often manifested in divergent growth trajectories, carries significant implications for the capacity of populations to adapt and respond to changing environmental conditions (Costa‐Pereira et al. 2019). Yet, variation in realized growth does not imply that individuals are free to express their full growth potential. Instead, growth is inherently constrained by fundamental life‐history trade‐offs among somatic growth, survival, and reproduction, which limit the allocation of energy across competing functions (Dmitriew 2011; Lancaster et al. 2017). So, realized growth trajectories emerge from the interaction between intrinsic differences among individuals and environmental variation, including temperature and resource regimes, which jointly shape energy allocation patterns and demographic outcomes (Caceres et al. 2026; Cozzoli et al. 2022; Neuparth et al. 2002; Shelton et al. 2013). While groups sharing similar resources are often characterized by high niche overlap—thereby ramping up intraspecific competition—such ecological similarity does not preclude marked genotypic and phenotypic heterogeneity.

Indeed phenotypic diversity persists under high intraspecific competition as competition can be strongest among individuals with similar phenotypes (Blake and Coulson 2023; Pianka 1983; Goodrich and Clark 2023; Shokri et al. 2024). In this sense, differences in performance among individuals facing similar stressors may reflect not only fixed trait differences, but also variation in physiological plasticity and metabolic regulation, which can influence energy allocation, growth trajectories, and ultimately population persistence under environmental change (Auer et al. 2015; Mathot and Frankenhuis 2018). Intraspecific trait variability, reinforced by fine‐scale environmental heterogeneity, further allows phenotypically distinct individuals to coexist within the population (Bolnick et al. 2003, 2011; Burton et al. 2011; Violle et al. 2012).

Although the ecological and evolutionary significance of such variability is increasingly recognized, especially in relation to population persistence under environmental change (Chown and Terblanche 2006; Mathot and Frankenhuis 2018; Maldonado‐Chaparro et al. 2017), our understanding of the underlying physiological and molecular mechanisms driving these individual differences remains limited. In particular, it is still unclear which differences among conspecific individuals in nutrient assimilation, energy allocation, maintenance costs, and biomass synthesis are sufficient to produce divergent growth trajectories (Sokolova 2021; Sokolova et al. 2012; Sokolova 2021; Glazier 2015; Sibly et al. 2015). Because growth emerges from the integration of these metabolic and bioenergetic processes, identifying the physiological differences that distinguish alternative growth phenotypes is essential for understanding how intraspecific variability is generated and maintained within populations (Bolnick et al. 2011; Violle et al. 2012). Given that growth is a complex trait integrating multiple physiological and biochemical processes, including nutrient acquisition, energy metabolism, and protein synthesis (Arendt 1997; Pettersen et al. 2018), exploring these anabolic factors is vital. Identifying the key determinants that differentiate fast‐growing from slow‐growing individuals can thus provide valuable insights into the mechanistic basis of growth variability and its potential for evolutionary change. In this context, individual‐level metabolomics offers a promising way to discriminate the biochemical signatures associated with such differences (Goode et al. 2020; Jones et al. 2013).

The emerging field of environmental metabolomics examines the interactions between organisms and their environment by analyzing the small molecules (metabolites) present either in environmental samples or within organisms experiencing specific conditions (Jones et al. 2013; Lankadurai et al. 2013). Such approaches not only deepen our understanding of how organisms biochemically respond to changes, stressors, pollutants, and other external factors, with applications ranging from ecosystem health monitoring to assessing climate change impacts (Peñuelas and Sardans 2009; Viant et al. 2003), but also integrate multiple metabolic profiling techniques, such as GC/LC–MS spectrometry and ^1^H NMR spectroscopy, alongside pattern recognition methodologies. These combined techniques provide a comprehensive “snapshot” of low molecular weight metabolites that can vary during disease, upon exposure to foreign substances, or throughout an organism's development (De Castro et al. 2019; Nicholson et al. 2002). A metabolomic approach serves as a functional link between genetic potential and phenotypic expression.

This study aims to identify the key metabolic pathways driving growth differences and trait heterogeneity within *Gammarus insensibilis* individuals (Sibly et al. 2015; Stock 1967). Identifying metabolic variations is crucial to mechanistically explain why individuals exhibit distinct growth trajectories. To our knowledge, up to date, no studies have applied an NMR‐based metabolomic technique to characterize the metabolic fingerprint of this species. The NMR‐based approach provides highly reproducible, quantitative, and non‐destructive profiling, which is particularly useful when working with single organisms characterized by small biomass. While MS‐based metabolomics offers higher sensitivity and broader metabolite coverage, the advantages offered by an NMR‐based approach in terms of extraction variability and quantification robustness were key considerations for the present study (De Castro et al. 2019; Emwas 2015).

We used a combined ecological and molecular approach focused on cohorts of individuals reared under controlled laboratory conditions to amplify intrinsic growth differences, minimizing the external sources of variability (Dmitriew 2011; Peacor et al. 2007; Sherman and Campbell 1935; Sibly et al. 2015). Moreover, in‐depth analyses of metabolic pathways using advanced omics techniques provide insights into the mechanisms underlying growth variability. These results clarify how anabolic processes drive individual growth variation, prompting further investigation into their role in adaptation and evolution.

Based on the divergent growth phenotypes observed, we hypothesize that the metabolomic profiles reflect distinct strategies in resource allocation. Specifically, fast‐growing individuals are expected to exhibit a streamlined metabolism characterized by high anabolic efficiency, evidenced by an optimized protein synthesis pathway. Conversely, slow‐growing individuals are hypothesized to show metabolic signatures of physiological trade‐offs, such as increased catabolic signaling, higher oxidative stress markers, or inefficiencies in energy partitioning.

## 2 Experimental Section

### 2.1 Objective Species

This study focused on the amphipod *Gammarus insensibilis* (Stock 1967), a widespread aquatic macroinvertebrate characteristic of sheltered, shallow lagoon ecosystems. The species exhibits marked ecological tolerance, withstanding salinity conditions ranging from freshwater to hypersaline environments and temperatures between 2°C and 28°C (Gates 2006). As a detritivore, it primarily feeds on decomposing organic matter and on lipid‐ and protein‐rich microfungi conditioning plant litter; however, when preferred resources are limited, individuals can exploit less palatable substrates (Arsuffi and Suberkropp 1989; Cozzoli et al. 2022; Mancinelli 2012; Rossi 1985). Consistent with this trophic flexibility, ingestion rates are strongly resource‐dependent and, when fed microfungi‐conditioned reed leaves, may range between 40% and 100% of body mass per day (Berezina 2007).

With a lifespan of approximately one year, *G. insensibilis* can reach an adult body length of up to 19 mm. Development is direct, and reproductive activity can begin at a body length of roughly 4.5 mm (Sheader 1996; Gates 2006), making the species particularly suitable for experimental studies linking growth, maturation, and metabolic traits across ontogeny. Indeed, populations have been extensively investigated under controlled laboratory conditions, and standardized protocols are available for both rearing procedures and the quantification of individual morpho‐functional traits (Shokri et al. 2024). For the present study, founding adults were collected from the coastal lagoon “Pantano Grande” within the Le Cesine (Salento peninsula, south‐eastern Italy), with the authorization for specimen collection by the competent authority (WWF, World Wildlife Fund for Nature, Italy). Experimental individuals consisted of offspring released under laboratory conditions from these field‐collected adults.

### 2.2 Laboratory Conditions

The individuals collected from “Pantano Grande” were transported to the laboratory. Upon arrival, specimens were maintained in aerated trays filled with field water for two days to allow recovery from handling stress. After 48 h, they were transferred to aquaria containing 40 L of water adjusted to salinity and temperature conditions representative of the long‐term annual mean values recorded at the collection site (10 PSU, 18°C). These conditions correspond to the typical environmental regime experienced by the species and were selected to reproduce ecologically realistic, non‐stressful maintenance conditions. Aquaria were continuously aerated and exposed to natural light conditions through laboratory windows, reflecting ambient photoperiod conditions (approximately corresponding to a 12 h light: 12 h dark). Temperature and salinity were kept constant throughout the experiment to minimize environmental variability among individuals. Animals were provided with an *ad libitum* amount of conditioned reed leaves ( *Phragmites australis* ) which represent a natural detrital resource for lagoonal amphipods. To reproduce this natural trophic pathway, reed leaves were conditioned for 15 days in aerated channels containing lagoon water enriched with naturally occurring microbial communities collected from the sampling site. This conditioning process promotes microbial colonization of the detrital substrate and increases its nutritional value, making it comparable to the food resources exploited by *G. insensibilis* in the field.

Ovigerous females were visually identified and isolated from the maintenance aquaria. Upon releasing their offspring, the females were individually transferred to separate cups containing 10 PSU water, an oxygenator, and an *ad libitum* supply of resources. Each day, the newly released offspring were counted and moved to trays labeled according to the day of release, where they stayed for 7 days. This procedure allowed individuals to pass the initial post‐release mortality phase, which is typically elevated during the first days after leaving the maternal brood pouch. A cohort of 250 individuals born within a three‐day interval was established. Individuals at the cohort starting time were at the 7th to 9th days old. The use of synchronously born individuals minimized ontogenetic variability among specimens and ensured that individuals experienced comparable developmental conditions during the experiment. The cohort was maintained in the experimental tray and provided with an ad libitum resource supply of 20 g of 15 days conditioned reed leaves. The overall health of the cohort population was monitored daily. Resources were replenished every two weeks with 20 g of 15 days conditioned reed leaves, in order to maintain consistent feeding conditions. After 45 days, during a scheduled resource renewal, individuals were sorted according to their body size into three size categories: Small (S), Medium (M), and Large (L). Every size class was then transferred to a new experimental tray. To maintain a consistent individual‐to‐resource ratio as that of their first 45 days, 0.08 g of conditioned reed leaves were provided for every individual in the new trays. This improved the interpretability of day‐75 differences in body size and metabolism as outcomes of divergent growth trajectories rather than ongoing interference among strongly size‐mismatched conspecifics. At the end of the 75 days, single individual body dimension, sex, and metabolism were assessed.

### 2.3 Individual Body Dimensions

The wet weight of each individual was measured. To avoid the excess of water, each animal was first placed on highly absorbent paper and then its weight measured using a six‐digit microanalytical balance (Sartorius MC5; +1 mg accuracy). Dry weight (DW) was estimated from wet weight (WW) using a species‐specific calibration equation derived from individuals maintained for 75 days under the same experimental conditions.

DW=0.25×W+0.15

(number of individuals = 34 *R* ^2^ = 0.82, RMSE = 0.35 mg).

Growth rate was calculated as the ratio between individual dry weight (DW) and the number of growth days. This metric represents a time‐averaged index of biomass accumulation over the experimental period rather than an assumption of strictly linear growth. Because all individuals were maintained under standardized conditions and within a comparable developmental window (~75 days), this integrated measure allows robust comparisons among size classes while avoiding additional model assumptions regarding growth trajectories. Following the weighing process, the length and sex of each animal were determined with a Nikon stereoscope. For measurement of body size and sex, individuals were first anesthetized in carbon‐dioxide‐saturated water and placed under a stereomicroscope (Nikon SMZ1270) equipped with a camera connected to a computer. Images were acquired and analyzed with Nikon NIS‐Elements software. Body length was measured following the natural contour of the body from the base of the antennae to the base of the telson using the dorsal margin of the body as a reference landmark (Basset and Glazier 1995). Sex was determined under the stereomicroscope based on external sexual characters. Females were identified by the presence of oostegites (brood plates) forming a marsupial pouch on the ventral side. Male assessment was supported by the presence of sexually dimorphic gnathopods, which in *Gammarus* species are typically enlarged in males and associated with reproductive behavior and copulation (Hume et al. 2005). After these measurements, a subset of the cohort (*n* = 36 individuals) was selected for metabolomic analyses: 15 individuals from the Small size class, 12 from the Medium size class, and 9 from the Large size class were individually placed in Eppendorf tubes and stored at −80°C.

### 2.4 *Gammarus* Work Up for NMR Analysis

The Gammarus individuals (*n* = 36), as described in section 2.3, were analyzed by using NMR spectroscopy analysis. The freezed collected samples were lyophilizated to remove the water content, and the entire obtained dried powder resuspended in 700 μL of D_2_O based phosphate buffer at fixed pH of 7.4 to reduce NMR chemical shifts variations, prepared containing 3‐(trimethylsilyl)propionic acid (TSP) as previously reported (De Castro et al. 2022). Samples were then sonicated for 5 min and centrifuged at 12,000 *g* for 5 min at 4°C to remove any solid debris, and 600 μL of the supernatant transferred in a 5 mm NMR tube (Nagato et al. 2013; Viant 2008). All handling steps were conducted on ice and the time between thawing and extraction was kept strictly under 5 min. As previously reported, metabolic quenching was performed using flash freezing and cold protein precipitation to limit enzymatic activity and the thermal degradation of sensitive metabolites (Lankadurai et al. 2013).

### 2.5 NMR Measurements

All measurements were conducted using a Bruker Avance III 600 Ascend NMR spectrometer (Bruker, Hamburg, Germany) functioning at 600.13 MHz for ^1^H detection, featuring a *z* axis gradient coil and automatic tuning‐matching (ATM). Experiments parameters were carried out as previously reported. In detail, ^1^H zgcppr experiments were acquired at 300 K in the automation mode, NS = 1024, SW = 20.0276, D1 = 5.00 s (De Castro et al. 2020, 2022). Metabolite assignment was initially performed based on 1D ^1^H zgcppr NMR spectra, using chemical‐shift information from publicly available databases (HMDB, BMRB) and literature data references (De Castro et al. 2020; Fan 1996). Peaks were assigned only when the experimental chemical shifts and multiplicity patterns were consistent with reference values. This approach minimizes the risk of biased or speculative identification. The assignments were further supported using Chenomx NMR Suite v12.01, which provides spectral fitting and deconvolution tools that help resolve partially overlapping resonances.

### 2.6 Data Analysis

NMR spectral data were analyzed using the software Topspin 3.6.1, supplemented by Amix 3.9.13 (Bruker, Biospin, Italy) to facilitate simultaneous visual examination and subsequent data bucketing. In this procedure, the proton (^1^H) NMR spectra of aqueous extracts were partitioned into uniform rectangular buckets, each with a fixed width of 0.04 ppm, and subsequently integrated. To prevent interference from the residual water resonance signal, the spectral region between 5.10 and 4.70 ppm was excluded from analysis. To account for variations in individual biomass during sample preparation, the remaining buckets were normalized to their total spectral area to address minor discrepancies in signal intensity. This approach standardizes each sample to a constant total intensity, mitigating confounding effects due to differences in dry weight. Further data scaling was applied using the Pareto method, whereby mean‐centered values were adjusted through division by the square root of their standard deviation, as detailed by Van Den Berg et al. (2006). This enhance the contribution of lower‐concentration metabolites without inflating noise. Multivariate statistical analyses were employed to explore intrinsic variations within the dataset, including unsupervised principal component analysis (PCA) and supervised orthogonal partial least squares discriminant analysis (OPLS‐DA), conducted via SIMCA 14 software (Sartorius Stedim Biotech, UmeÅ, Sweden) in accordance with the methodology outlined by Bro et al. (2008). The reliability and predictive performance of the statistical models developed for group discrimination were scrutinized using a default cross‐validation approach (7‐folds) and further evaluated through permutation testing with 100 iterations, consistent with established protocols (Bro et al. 2008). The quality and explanatory power of the statistical models were assessed using cumulative *R* ^2^ (*R* ^2^(cum)) and cumulative *Q* ^2^ (*Q* ^2^(cum)) metrics alongside statistical significance values (*p*[CV‐ANOVA]) obtained through cross‐validated predictive residual analysis (CV‐ANOVA), where *p*‐values < 0.05 (corresponding to a 95% confidence level) were deemed statistically significant. Pairwise group comparisons were subjected to two‐sample *t*‐tests, with statistical variance considered significant at *p*‐values < 0.05. For metabolites identified as statistically significant through ANOVA and complementary post hoc analyses, box‐and‐whisker plots were generated to illustrate variation among experimental replicates and conditions, including differences associated with varying body sizes. These visualizations enabled a more comprehensive understanding of metabolite behavior under distinct experimental scenarios.

### 2.7 Metabolic Pathway Analysis

The MetaboAnalyst software (Chong et al. 2018) was employed to identify altered metabolic pathways through pairwise comparisons. To maximize the differences, the analysis focused on Small and Large individuals. For the metabolic pathway analysis, previously quantified (using selected distinctive unbiased NMR signals) highly significantly altered metabolites of Small and Large individuals (tyrosine, glucose, glycine, TMAO, methionine, glutamate, 2‐aminobutyrate, alanine, lactate, lipids, valine) were utilized as the input matrix. A statistical threshold such as *p*‐value (*p* < 0.05) and impact value was applied. The pathway impact was determined as the sum of the importance measures of the matched metabolites, normalized by the total importance measures of all metabolites within each pathway (Chong et al. 2018). Since *Gammarus insensibilis* is not present in the Metabo Analyst Pathway Analysis (MeTPA) database, *Daphnia pulex* , the most studied and closely related crustacean species available in the MetaboAnalyst, was used as a reference for the pathway investigations. Its use as a reference for *Gammarus insensibilis* is a justified compromise. Core metabolic pathways are highly conserved across freshwater invertebrates, and species‐specific differences, typically related to detoxification (Campos et al. 2016), are unlikely to bias this study, as no chemical exposure was involved.

## 3 Results

### 3.1 Individual Growth Rate

**FIGURE 1:**
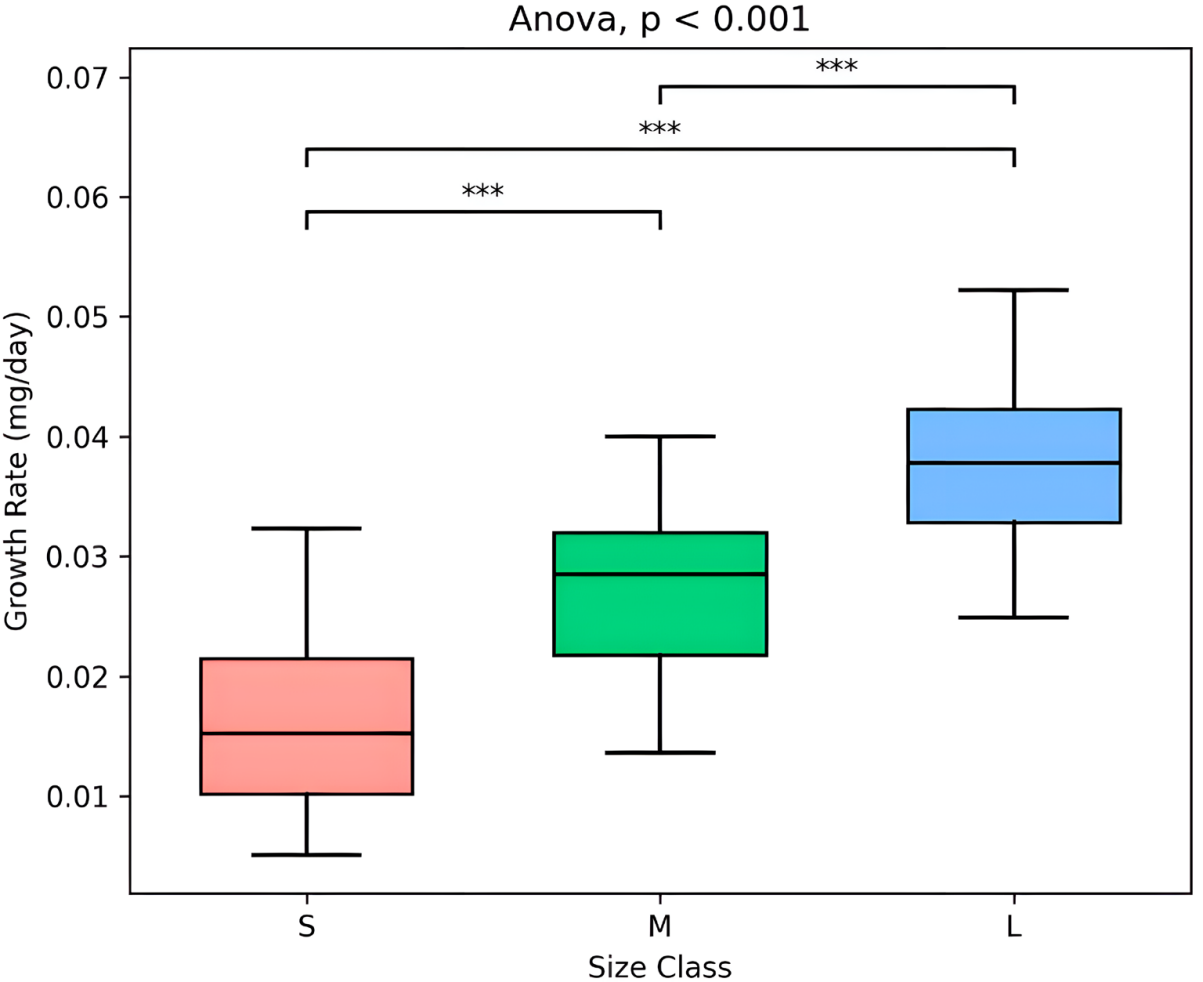
Box and whisker plot displaying the daily growth rate of the *Gammarus insensibilis* individuals grouped according to their size class (Small, in red, Medium, in green, and Large, in blue). A total of *n* = 36 individuals was selected: 15 individuals from the Small size class, 12 from the Medium size class, and 9 from the Large size class.

After 75 days, the body sizes of *Gammarus insensibilis* mirrored the pattern observed at the 45‐day resource renewal. Individual growth rate was estimated as the ratio of final dry weight to experimental duration (75 days). A one‐way ANOVA revealed significant differences in growth rate and dimensions among the three size classes (*F*_2,83_ = 61.91, *p* < 2 × 10^16^) with individuals of the Small size class showing a mean dry weight of 1.26 ± 0.62 mg and a mean daily growth rate of 0.016 ± 0.010 mg·day^−1^ (*n* = 32). Individuals in the Medium class averaged 2.12 ± 0.60 mg in dry weight with a growth rate of 0.028 ± 0.010 mg·day^−1^ (*n* = 28), while those in the Large class reached 2.81 ± 0.57 mg with a daily growth rate of 0.038 ± 0.010 mg·day^−1^ (*n* = 26), Figure 1.

### 3.2 NMR Characterization of *Gammarus insensibilis*

**FIGURE 2:**
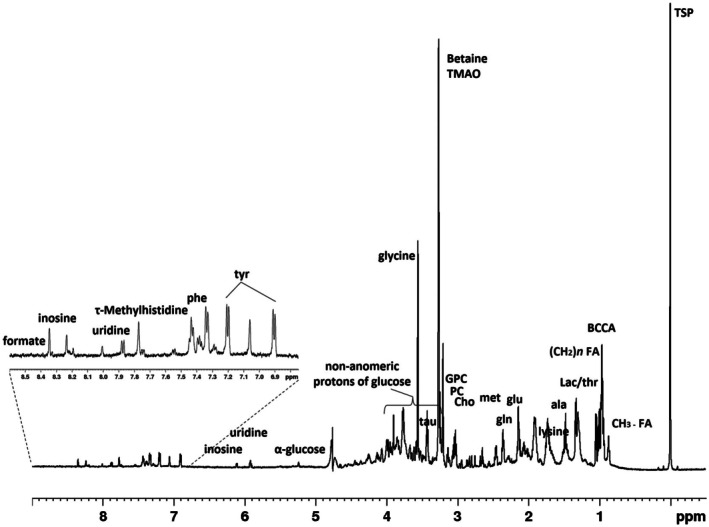
Representative ^1^H NMR spectrum for sample aqueous extract (D_2_O, 600 MHz NMR spectrometer). BCCA, Branched chain aminoacids (valine, leucine, isoleucine); Cho, Choline; FA, Fatty acids; FA, Fatty acids; gln, Glutamine; glu, Glutamate; GPC, Glycerophosphocholine; lac, Lactate; thr, Threonine; met, Methionine; phe, Phenylalanine; PC, Phosphocholine; tau, Taurine; tyr, Tyrosine.

In the present research, the metabolic profile of *Gammarus insensibilis* aqueous extracts was characterized by using NMR spectroscopy. The representative one‐dimensional ^1^H zgcppr NMR spectrum, Figure 2, and the primary resonances were assigned to individual metabolites and respectively reported in Table 1.

**TABLE 1:** Chemical shifts (δ) and assignments of metabolite resonances in the *Gammarus Insensibilis* ^1^H NMR spectrum.

Metabolites	^1^H NMR chemical shift (δ, ppm)
2‐aminobutyrate	0.97(t); 1.92(m); 3.75 (t)
Betaine	3.27(s); 3.91 (s)
Choline	3.19(s); 3.52(m); 4.07(m)
Formate	8.46(s)
Glycerophosphocholine	325(s); 3.81(m); 4.30(m)
Glycine	3.56(s)
Glutamate	2.10 (m), 2.35 (m), 3.70 (dd)
Glutamine	2.14(m); 2.46(m); 3.78(t)
Inosine	6.10 (d); 8.24(s) 8.36(s)
Isoleucine	0.94(t); 1.01(d); 1.26(m); 1.46(m); 1.98(m); 3.67(d)
Lactate	1.33(d); 4.12(q)
Leucine	0.96(d); 0.97(d); 1.70(m); 3.75(m)
Lysine	1.46(m); 1.73(m); 1.91(m);3.03(t); 3.76(t)
Methionine	2.14(s); 2.16(m); 2.65(t); 3.86(t)
Phenylalanine	3.13(m); 3.28(m); 4.00(m); 7.33(d); 7.38(t); 7.43(m)
Phosphocholine	3.20(s); 3.53(m); 4.14(m)
Taurine	3.25(t); 3.43(t)
Threonine	1.33(d); 3.59(d); 4.25(m)
TMAO	3.29(s)
Tyrosine	3.06(m); 3.20(m); 3.94(m); 6.92(d)*; 7.20(d)
Uridine	5.91(d); 7.84
Valine	0.99(d); 1.04(d); 2.28(m); 3.61(d)
α‐Glucose	3.42(t); 3.54(dd); 3.71(t); 3.74(m); 3.84(m); 5.24(d)
β‐Glucose^(C,M)^	3.25(dd); 3.41(t); 3.46(m); 3.49(t); 3.72(dd); 3.90(dd); 4.65(d)
τ‐methylhistidine	7.06 (s); 7.76 (s)
C*H*_*3*_ of all f.a.	0.88(t)
f.a chain length (CH_2_)*n*	1.28(m)

*Note:* Betaine (N‐trymethilglycine), f.a.: fatty acids. TMAO (N‐trymethilamine oxide). Letters in parentheses indicate the peak multiplicities; d, doublet; dd, doublet of doublet; m, multiplet; q, quartet; s, singlet; t, triplet.

A total of 25 metabolites were identified and compared with previously reported data (Chiu et al. 2017; De Castro et al. 2022; Fan 1996).

Consistent with the metabolic demands of growth and biomass accumulation, the qualitative analysis of the *Gammarus insensibilis* extracts NMR spectra reveals key metabolic precursors. These are dominated by amino acids essential for protein synthesis (isoleucine, leucine, valine, glycine, alanine, phenylalanine, tyrosine, glutamate, glutamine, lysine, methionine, and τ‐methylhistidine), alongside organic acids (lactate, 2‐aminobutyrate) and nucleosides (uridine, inosine) involved in energy pathways and nucleotide turnover. This growth‐oriented profile is further characterized by a specialized set of osmolytes—including TMAO, taurine, and betaine—and lipid signals ((CH_2_)_n_ and CH_3_), highlighting a significant metabolic investment in both homeostasis and structural development.

### 3.3 Multivariate Data Analysis

**FIGURE 3:**
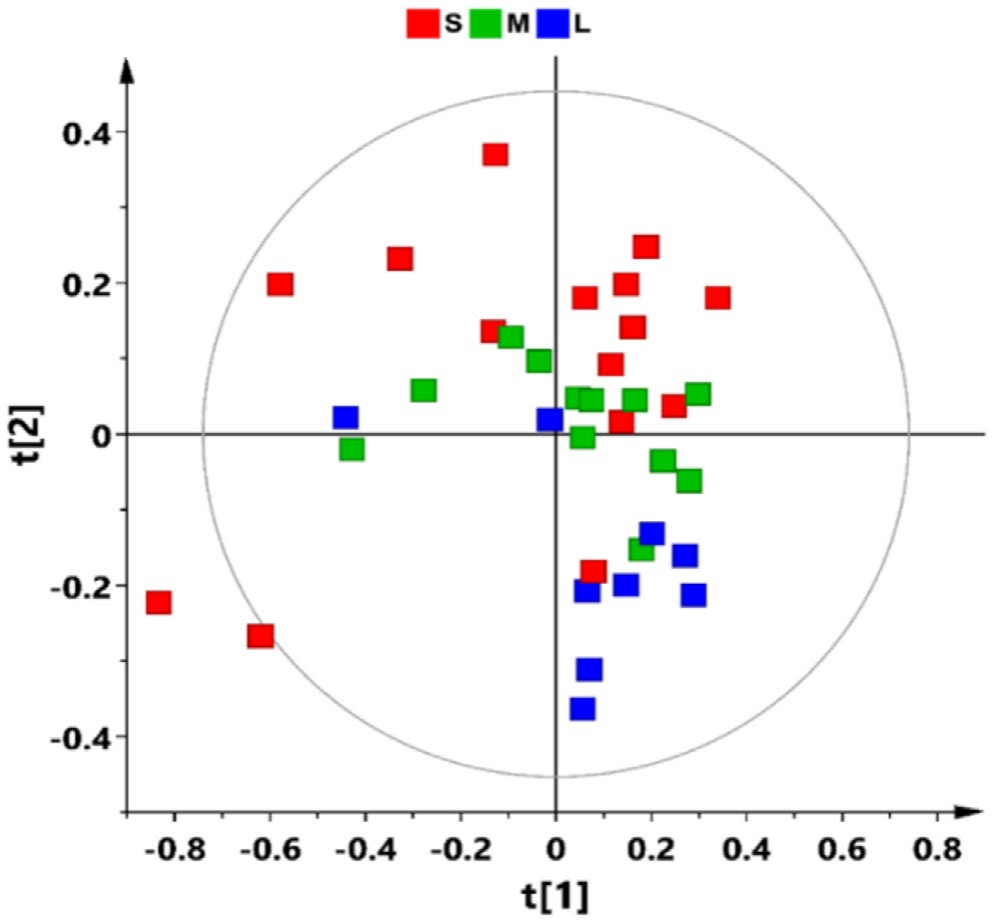
PCA (5 components; R^2^X(cum) 0.767, R^2^Y(cum) 0.418) score plot of all analyzed samples.

Multivariate statistical analyses were applied to the whole dataset to assess differences in metabolic profiles among the sample groups relating to Small (S), Medium (M), and Large (L) sized individuals for a total of 36 samples. To explore natural groupings of the samples without a prior definition of their class membership, a preliminary unsupervised Principal Component Analysis (PCA) was performed, Figure 3. A five‐component PCA model describes 76.7% of the total variance of the dataset. Considering the PC1 and PC2 of the model (37.5% and 14.1% of the total variance respectively), Figure 3, it was observed that the samples cluster along the t[2] component of the model according to the increasing size of Gammarus, showing changes in metabolite composition and/or levels of expression in relation to the dimension of individuals. A certain degree of separation was observed among the “small” individuals, with two samples deviating from the general data distribution visible as outliers in the considered model.

**FIGURE 4:**
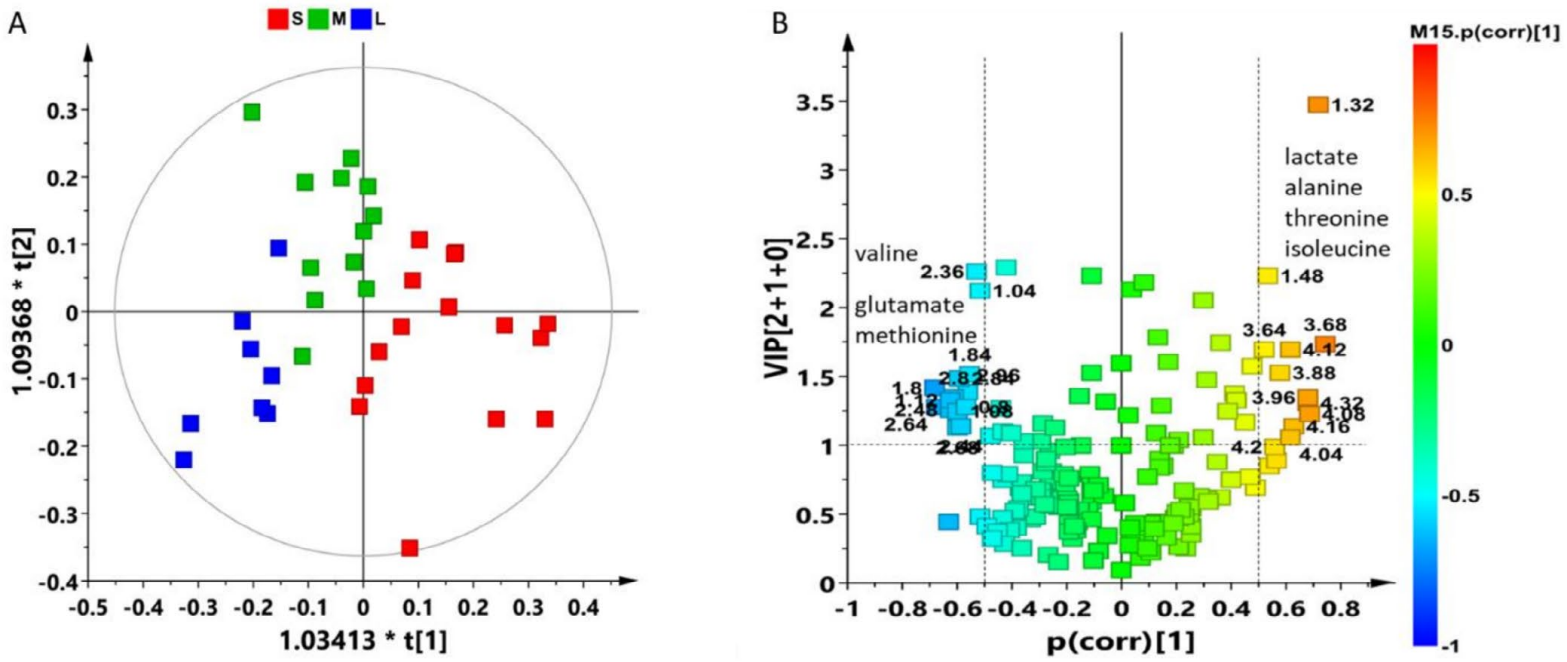
(A) The OPLS‐DA score plot (model parameters: 2 + 1 + 0, R^2^X(cum) = 0.585, R^2^Y(cum) = 0.554, *Q* ^2^(cum) = 0.343) derived from ^1^H NMR aqueous extracts, visually describes the separation of *Gammarus insensibilis* samples based on their metabolic profiles. (B) The corresponding relative Volcano plot for the model illustrates the predictive loadings, utilizing Variable Importance in Projection (VIP) scores and *p*(corr) values. Variables are color‐coded based on correlation‐scaled loading (*p*(corr)) values from the dataset, with numbers representing chemical shifts (ppm) from the ^1^H NMR spectra. Additionally, the identified metabolites are clearly labeled for reference.

By applying supervised statistical methods, a well descriptive but weakly predictive OPLS‐DA model (2 + 1 + 0, R^2^X(cum) = 0.585, R^2^Y (cum) = 0.554, *Q* ^2^ (cum) = 0.343) was obtained for the 36 samples analyzed, Figures 4A and Table S1. The outliers identified in the first two components of the PCA model, Figure 3, were not excluded. As this is a single‐organism analysis, and these individuals do not behave as outliersconsidering also the other components of the PCA model, their inclusion was deemed appropriate for a comprehensive representation of the data in the OPLS‐DA supervised model.

The Small (S) individuals were distinctly separated from the Large (L) ones along the predictive t[1] component. The Medium (M) samples are grouped at the middle of the other two classes (S and L) along the predictive t[1] component. Furthermore, a separation along the t[2] orthogonal component was also appreciable for these latter.

The analysis of the variables, represented by NMR signals, that contribute to class separation was conducted through the analysis of the corresponding Volcano Plot, illustrated in Figure 4B. This plot presents the predictive loadings by integrating the Variable Importance in Projection (VIP) scores with the corresponding *p*(corr) values.

This evaluation revealed that S samples exhibited a high relative content of lactate, alanine, threonine, and isoleucine. Conversely, L and M samples were characterized by a high relative content of valine, glutamate, and methionine.

**FIGURE 5:**
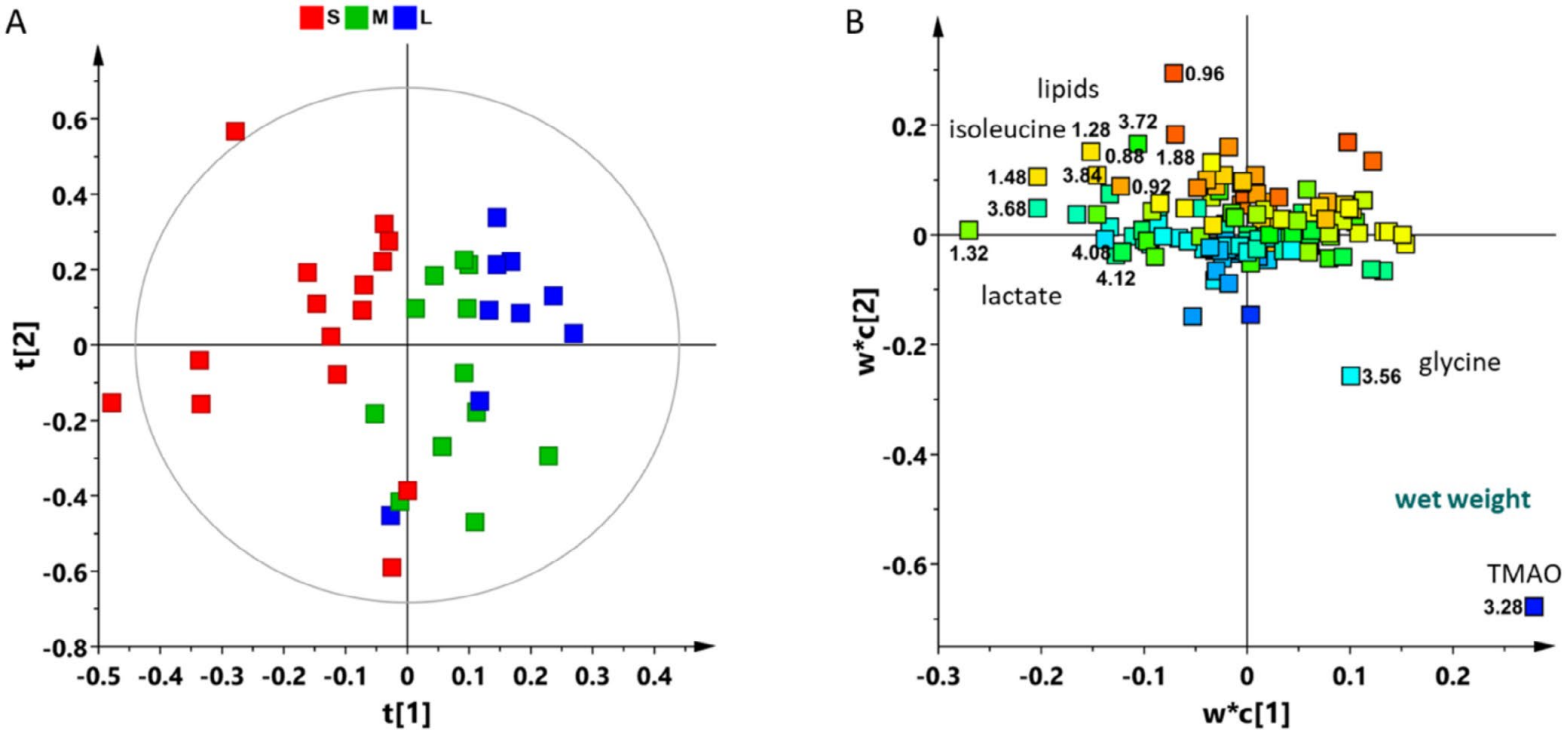
(A) PLS regression model score plot (2 components, R^2^X(cum) = 0.482, R^2^Y (cum) = 0.542, *Q* ^2^ (cum) = 0.25) score plot obtained from ^1^H NMR aqueous extracts for *Gammarus Insensibilis* and (B) relative loading plot. Y variables = size; wet weight (mg).

In the further Partial Least Squares (PLS) regression model (2 components, R^2^X(cum) = 0.482, R^2^Y(cum) = 0.542, *Q* ^2^(cum) = 0.25), Figure 5, the predictor variables (loadings) were related to response variables (size, wet weight). The loading plot, Figure 5B, displayed how the predictor variables contribute to the PLS components. The axes (w*c[1] and w*c[2]) represented how strongly each variable is correlated with the first two PLS components highlighting that the higher metabolic rate and wet weight were associated with the higher amount of glycine and TMAO in individuals with medium and large size with respect to the smalls characterized by a higher content of isoleucine, lipids and lactate (and lower wet weight).

**FIGURE 6:**
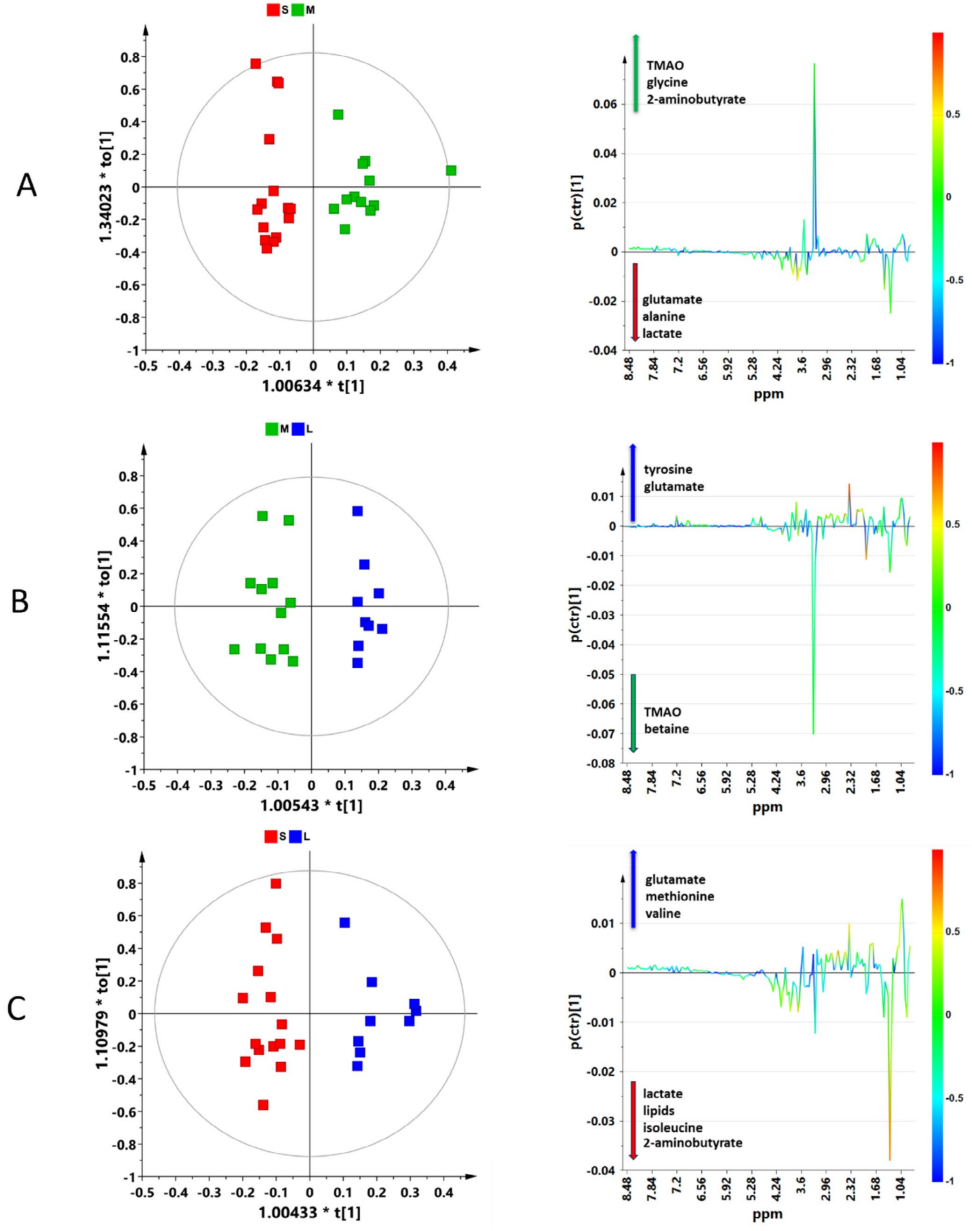
OPLS‐DA score plots (left panels) and the corresponding color‐coded correlation coefficient S‐line plots (right panels) illustrate the predictive discriminant loading variables derived from ^1^H zgcppr NMR spectra of *Gammarus insensibilis* , analyzed across different pair‐wise groups. Each of the OPLS‐DA models was built with one predictive and three orthogonal components (1 + 3 + 0) ensuring comparability across results. (A) Small versus Medium; R^2^X(cum) = 0.665, R^2^Y(cum) = 0.827, *Q* ^2^ = 0.332. (B) Medium versus Large; R^2^X(cum) = 0.722, R^2^Y(cum) = 0.918, *Q* ^2^ = 0.282. (C) Small vs. Large; R^2^X(cum) = 0.691, R^2^Y(cum) = 0.879, *Q* ^2^ = 0.513.

To maximize the observed class differences and identify the metabolites contributing to class separation, pairwise comparison OPLS‐DA models were carried out for the three conditions under consideration (S, M, L), as shown in Figure 6.

Small individuals are characterized by a higher content of glutamate, alanine and lactate in comparison with the Medium (Figure 6A right panel), and of lactate, lipids, isoleucine and 2‐aminobutyrate in comparison with that Large (Figure 6C right panel). The samples of Medium size, on the other hand, presented a higher content of TMAO, glycine, and 2‐amino butyrate with respect to the Small (Figure 6A right panel) and TMAO and betaine with respect to the Large (Figure 6B right panel). For Large samples, a higher content of glutamate, methionine, and valine with respect to Small (Figure 6C right panel) and tyrosine and glutamate in comparison with the Medium (Figure 6B) were found.

**FIGURE 7:**
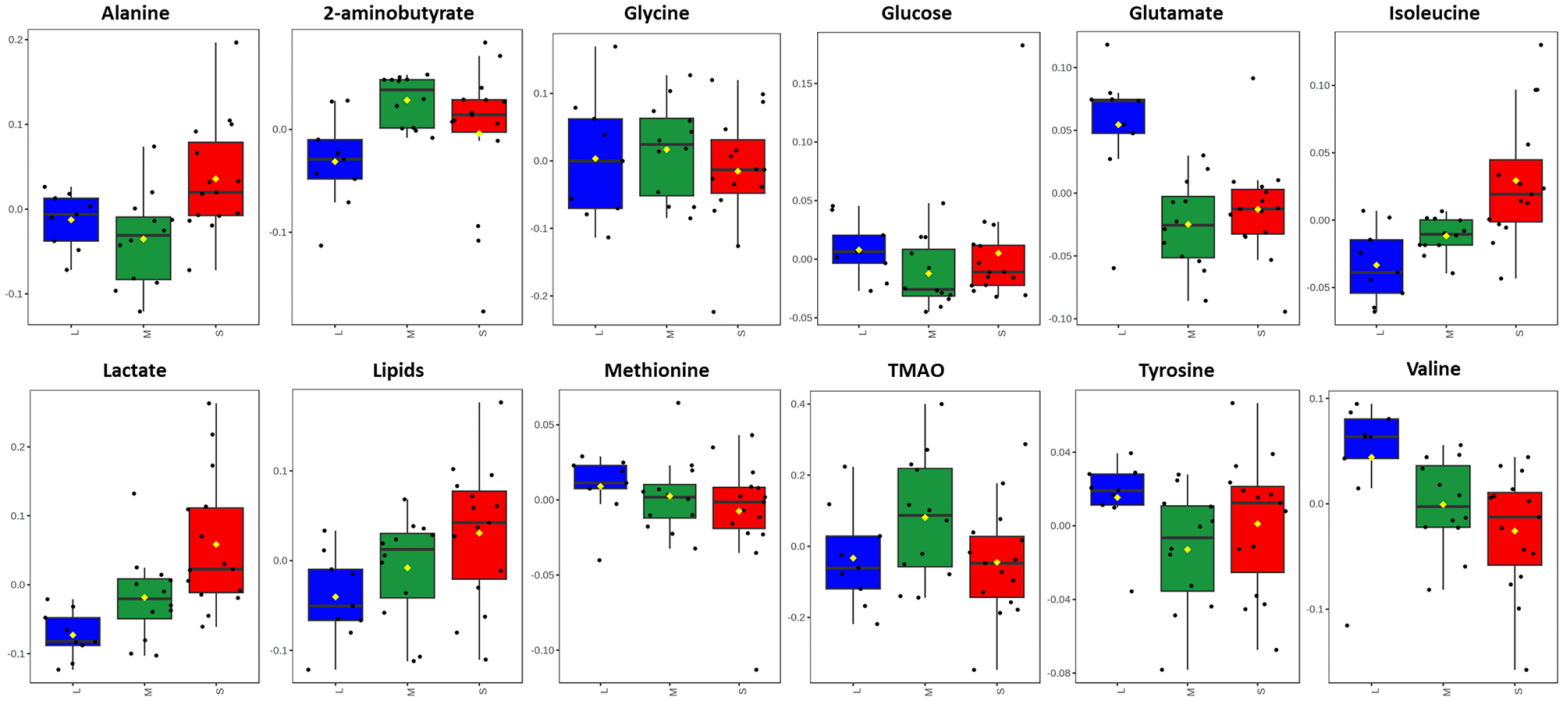
Box‐and‐whisker plots illustrate the distribution of the chemically identified markers that differ among the Small (red), Medium (green), and Large (blue) samples. These plots provide a summary of the normalized bucket values for each group. A yellow diamond represents the mean value within each group, while the notches signify the 95% confidence interval surrounding the group medians. Any dots beyond the whisker boundaries indicate outliers in the dataset.

The Box and whisker plots, Figure 7, illustrate the trends of alteration of the significant metabolites identified in the pairwise comparison OPLS‐DA models, Figure 6, upon individuals of Small, Medium, and Large size. The further carried out One‐way ANOVA with Tukey's post hoc test unveiled isoleucine, glutamate, and lactate as highly significant metabolites (*p* < 0.05).

### 3.4 Metabolic Pathway Analysis

**FIGURE 8:**
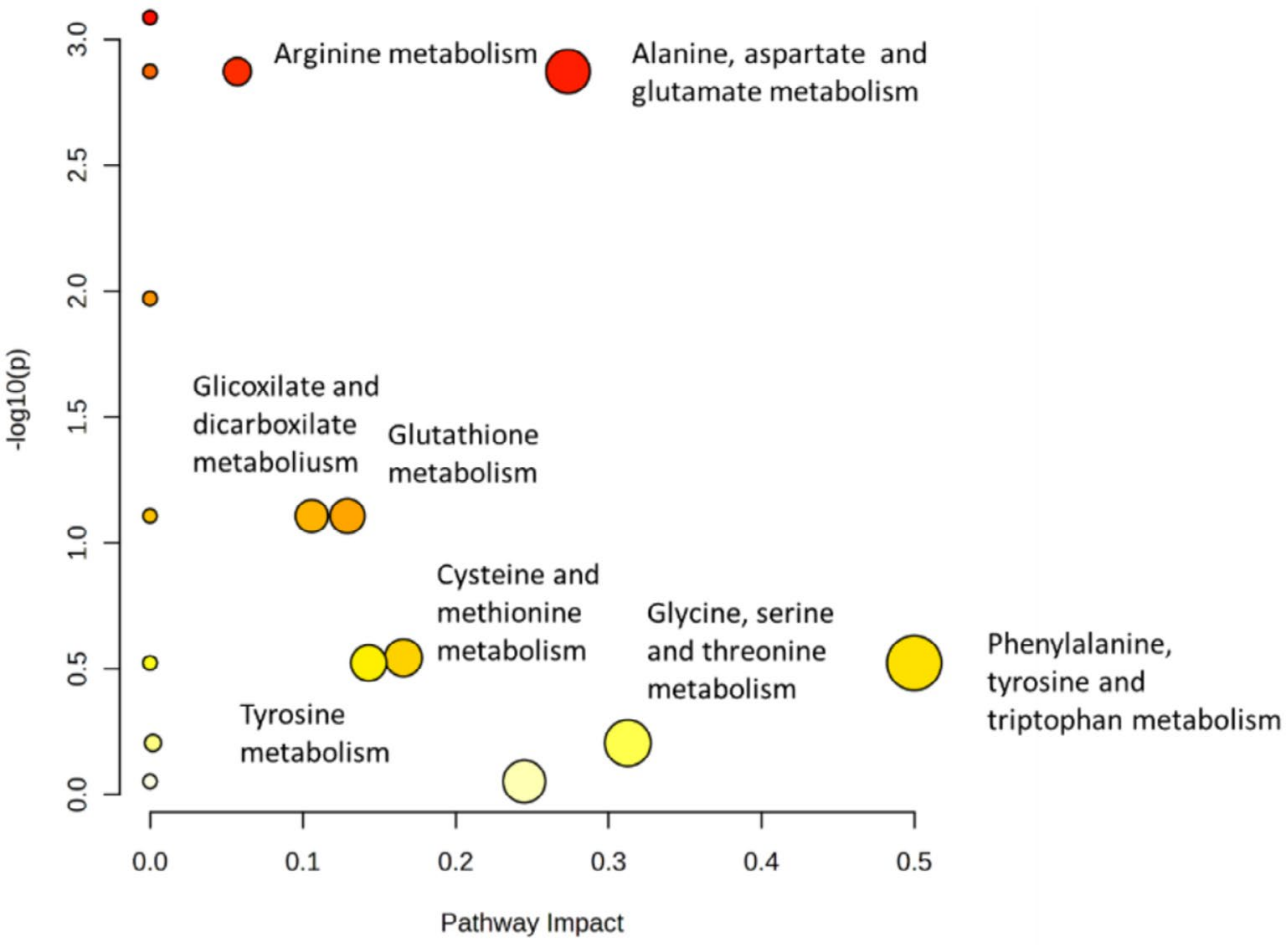
A pathway analysis was conducted to examine class size variations (Small vs. Large) using the library of *Daphnia pulex* . The visual representation employs a color gradient ranging from yellow to red, where red indicates metabolites with a statistically significant presence (*p*‐value < 0.05). Furthermore, the size of the circles corresponds to the degree of influence these metabolites exert on pathway alterations; smaller circles denote minimal impact, while larger circles signify higher levels of pathway perturbation.

Metabolic pathway analysis focused on Small and Large individuals, as they exhibited the most pronounced metabolic differences. The pathway analysis highlighted a small set of metabolic routes that differed clearly between the two groups. Among these, alanine, aspartate, and glutamate metabolism and arginine metabolism showed the strongest combination of statistical significance and pathway impact, emerging as the most altered pathways (Figure 8). Ribes‐ Navarro et al. demonstrated that amino‐acid metabolism in Gammarids is highly plastic and responds rapidly to energetic and nutritional demands (Ribes‐Navarro et al. 2022). Additional routes such as glutathione metabolism, glycine, serine and threonine metabolism, and aromatic aminoacid related pathways appeared with lower impact values, indicating more moderate alterations. Recently, it was demonstrated by some of us that larger gammarids show greater metabolic variability and stress sensitivity, consistent with activation of oxidative‐stress pathways such as the evidenced gluthathione and glicoxilate and dicarboxilate metabolism (Shokri et al. 2019).

## 4 Discussion and Conclusion

Understanding within‐population variation in life‐history strategies is central to Ecology and Evolution because it shapes how populations persist and respond to environmental change through differences among individuals in performance and trait trajectories (Park and Wootton 2021). In this study, we provide an NMR‐based metabolic characterization of *Gammarus insensibilis*, which looks to be the first metabolic description for the species, addressing intrapopulation clustering at the individual level. Because metabolites sit immediately downstream of gene expression and upstream of phenotype, their profiles can clarify mechanisms behind life‐history variation, hence testing whether individual differences in metabolic phenotype are associated with variation in growth rate within a single population (Balashova et al. 2022).

Overall, this approach links metabolite‐level variation to ecologically meaningful differences in growth, helping bridge physiology and life‐history variation within populations.

Multivariate analysis of the ^1^H NMR profiles revealed a clear metabolic differentiation among *G. insensibilis* size classes, consistent with their divergent growth trajectories. OPLS‐DA models discriminating Small (S), Medium (M), and Large (L) individuals indicated a metabolic reorganization associated with size‐dependent growth performance. Robust predictive performance (*Q* ^2^ values) is achieved only for the S vs. L individuals' comparison, Figure 6C, which showed a pronounced separation between the two groups, whereas the models involving the M group present expected statistical limitations that align with the biological characteristics of the system. Medium individuals (M) displayed intermediate metabolic features partially overlapping with both S and L individuals, thereby reducing the likelihood of achieving a clear separation and a robust predictive model.

Individuals in the Small size class, corresponding to slower‐growing phenotypes, showed relatively higher levels of lactate, alanine, threonine, and isoleucine, suggesting enhanced reliance on glycolytic flux and amino acid catabolism. In contrast, Medium and Large individuals, reflecting faster growth rates, were characterized by higher relative abundances of valine, glutamate, and methionine, metabolites more closely linked to anabolic processes, nitrogen metabolism, and biosynthetic capacity.

Together, these patterns indicate that size classes are not merely morphological categories but represent metabolically distinct phenotypes aligned with differential growth allocation strategies.

Aquatic crustaceans rely on a combination of aerobic and anaerobic pathways for energy production (Henry et al. 1994), the latter often characterized by lactate accumulation in muscle tissue and haemolymph (Sneddon et al. 1998; Walsh 1994). In our study, the elevated lactate levels observed in smaller individuals suggest a potentially greater reliance on glycolytic activity, which could be hypothesized as a strategy to support a high metabolic intensity during early development. This trend is consistent with higher alanine levels; however, while alanine is involved in energy metabolism and gluconeogenesis, its elevation could also reflect broader amino acid turnover or stress‐related responses. These metabolic signatures suggest that smaller individuals may prioritize rapid energy mobilization. In contrast, larger individuals exhibited a distinct profile characterized by higher glutamate levels. Rather than reflecting a ‘balanced’ metabolism, this may indicate a shift toward enhanced nitrogen metabolism and amino acid interconversion. While glutamate is a known neurotransmitter precursor, its prevalence here more likely reflects its central role in the nitrogen cycle and energy homeostasis (Andersen 2025; Foa and Cooke 1998). These size‐dependent differences highlight how metabolic strategies scale with body mass and developmental stage in *Gammarus insensibilis*. At the same time, additional analyses did not detect significant relationships between sex and growth rate, hence suggesting that sex is unlikely to account for the main metabolic structure observed, which was instead more consistently associated with growth‐related developmental differentiation among individuals.

The PLS regression model, in which the predictor variables (loadings) were related to response variables (wet weight across size classes), highlighted that individuals with larger body size (Medium and Large size) correlate with a higher amount of glycine and TMAO with respect to the smaller ones that correlate with a higher content of lipids and lactate (and related lower wet weight). The observed increase in glycine and TMAO in Medium and Large individuals may reflect a shift in osmotic regulation (Rodrigues et al. 2025). However, as salinity stress and osmotic pressure were not directly measured, this interpretation remains speculative for *G. insensibilis* . Drawing a comparative analogy from marine fish literature, TMAO is known to serve as a crucial osmotic regulator and protein stabilizer (Yancey et al. 2014). While its role in counteracting hydrostatic pressure or urea‐induced stress is well documented in other taxa (Liu et al. 2022), the ecological relevance of such adaptations, particularly regarding pressure, may be limited for shallow‐water amphipods. Furthermore, while it is intuitive to suggest that larger individuals possess greater muscle mass and metabolic activity requiring protein stabilization, this remains a hypothesis pending future morphometric or histological verification.

Regarding lipids, the primary organic reserve in many crustaceans (Shunmugam et al. 2022), our findings showed that lipid‐related NMR signals were more pronounced in Small individuals. While this elevated lipid content could reflect a specific energy allocation strategy, its interpretation remains complex. The presence of higher lipid levels alongside elevated lactate in smaller individuals suggests a multifaceted metabolic profile: while lactate may point toward significant glycolytic activity, the lipid abundance might represent stored reserves rather than active utilization (Jiang et al. 2020). On the other hand, fast‐growing individuals may exploit more readily available carbohydrates or proteins, benefiting from their ability to prioritize these substrates (Cormier et al. 2020; Morgan et al. 2002; Post and Parkinson 2001). Although individuals were reared under identical external resource conditions, previous studies have reported individual differences in feeding performance and competitive resource access in gammarid amphipods (Agatz and Brown 2014; Dickey et al. 2025). Further investigation is therefore needed to clarify how substrate mobilization influences growth rates in *Gammarus insensibilis* . Their ability to prioritize these faster‐metabolizing energy sources stems from a competitive edge in securing and processing nutrients, crucial for meeting the high metabolic demands of rapid growth.

**FIGURE 9:**
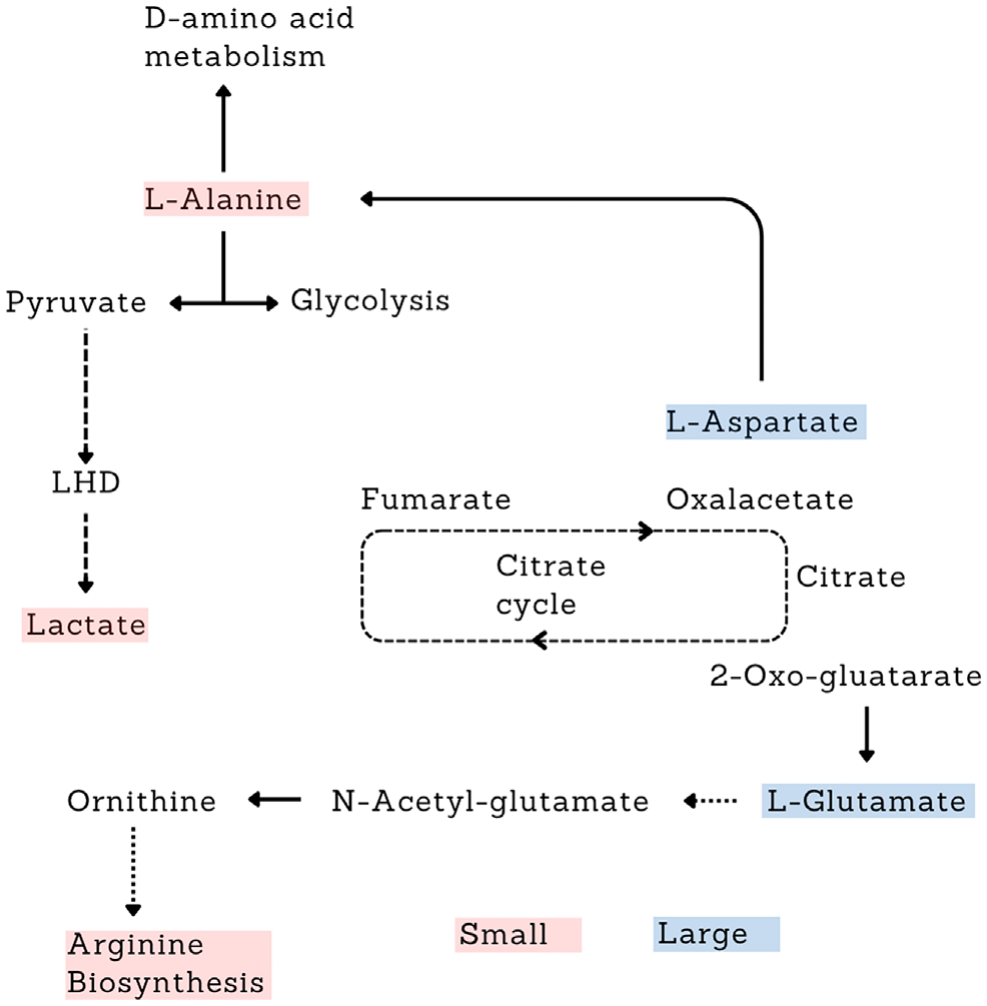
Schematic representation of the main pathways resulted altered between the Small (S) and Large (L) individuals from the performed pathway analysis. Red indicates higher amount in Small with respect to Large individuals. Blue indicates higher amount in Large with respect to Small individuals.

The pathway analysis identified at least two potential target metabolic pathways that are significantly altered between the Small and Large size classes (Figure 8), namely Alanine, Aspartate, and Glutamate metabolism, and Arginine metabolism (summarized in Figure 9). While these findings provide a biological framework for the observed differences, it is important to note that the pathway mapping relied on *Daphnia pulex* as a surrogate species due to current database limitations, which may affect the biological specificity of the inferred pathways. In individuals of the Large class, the involvement of these pathways, confirmed by the evidenced significant variations in individual metabolites such as alanine and glutamate, suggests a metabolic profile associated with distinct nutrient processing. Specifically, the Alanine, Aspartate, and Glutamate metabolism pathway represents a plausible mechanism for supporting the synthesis of amino acids foundational to protein production.

We acknowledge that the number of individuals analyzed was limited; however, NMR‐based metabolomics studies in ecological systems frequently rely on multivariate metabolic profiling to detect organism‐level physiological shifts rather than population‐level parameters, allowing mechanistic insights to emerge even from relatively small experimental datasets (Bundy et al. 2009; Nagato et al. 2013; Powers et al. 2024; Watanabe et al. 2015). Similarly, the limited number of metabolites detected reflects the typical coverage of ^1^H NMR for small aquatic invertebrates, where only relatively abundant compounds fall above the detection threshold. This is particularly evident in the present case, since we performed a single‐organism metabolomic analysis. *Gammarus insensibilis* individuals have a very small biomass, which restricts the absolute metabolite concentration available in the NMR tube. This naturally reduced the number of low abundant metabolites detected. NMR analysis prioritizes the high reproducibility and structural information over depth of coverage and gives quantitative robust information about the metabolites. Thus, this approach is particularly convenient for screening biochemical responses, such as those we investigated in *Gammarus insensibilis* . Future studies with larger sample sizes and complementary analytical approaches would help extend metabolite coverage and further strengthen the generality of these findings.

In conclusion, metabolomic analysis provided valuable information on metabolic differentiation among specimens of the same cohort of *Gammarus insensibilis* , with individuals differing in final body size achieved also exhibiting distinct biochemical profiles. Our findings suggest that these size class metabolic pathways differences are consistent with variation in physiological strategies associated with growth performance. Smaller individuals displayed metabolite signatures suggestive of greater investment in maintenance and homeostatic processes, whereas larger individuals exhibit signatures likely reflecting increased protein synthesis and turnover. Together, these results indicate that metabolic configuration may covary with individual growth outcomes within populations. Beyond these biological interpretations, this study underscores the value of individual‐level ^1^H NMR‐based metabolomics for investigating intraspecific physiological heterogeneity in *G. insensibilis* . By capturing metabolic variation at the scale of single organisms, this approach provides a mechanistic entry point for exploring how biochemical diversity may contribute to life‐history variation and broader ecological and evolutionary processes. Further studies will be needed to confirm whether these changes represent a programmed adaptive strategy.

## Author Contributions

**Federica De Castro:** formal analysis (lead), investigation (lead), methodology (equal), software (lead), validation (equal), visualization (equal), writing – original draft (equal), writing – review and editing (equal). **Ludovico Lezzi:** conceptualization (equal), data curation (lead), formal analysis (equal), investigation (lead), visualization (equal), writing – original draft (equal), writing – review and editing (equal). **Milad Shokri:** conceptualization (equal), data curation (equal), formal analysis (equal), investigation (equal), writing – review and editing (equal). **Laura Del Coco:** formal analysis (equal), investigation (equal). **Francesco Paolo Fanizzi:** resources (equal), supervision (equal), writing – review and editing (equal). **Alberto Basset:** conceptualization (equal), funding acquisition (lead), resources (equal), supervision (equal), writing – review and editing (equal).

## Funding

This work was supported by National Biodiversity Future Center—NBFC, CUP C63C22000520001, Project code: CN_00000033, and by ITINERIS (Italian Integrated Environmental Research Infrastructures System), Project code: IR0000032, both funded by the European Union–NextGenerationEU under the National Recovery and Resilience Plan (NRRP).

## Conflicts of Interest

The authors declare no conflicts of interest.

## Supporting information


**Figure S1:** Permutation test (*n* = 100) for the OPLS‐DA model of Figure 4.
**Table S1:** CV‐ ANOVA of the OPLS‐DA model of Figure 4.

## Data Availability

The background data are available on Zenodo https://doi.org/10.5281/zenodo.17079053.
