# Supplementary material for: Intrapopulation Metabolic Variation Reflects Growth Differences: A Cross‐Sectional Study on Gammarides
Source: Ecol Evol. 2026 Apr 29;16(5):e73544. doi: 10.1002/ece3.73544 (PMC13125960; doi:10.1002/ece3.73544)
Supplement: Supplementary file 1 — Figure S1: Permutation test (n = 100) for the OPLS‐DA model of Figure 4. Table S1: CV‐ ANOVA of the OPLS‐DA model of Figure 4. [file ECE3-16-e73544-s001.docx]

**Supplementary Information**

**Intrapopulation metabolic variation reflects growth differences:**

**a cross-sectional study on Gammarides**

Federica De Castro^1,2^*, Ludovico Lezzi^1^*, Milad Shokri^1,2^, Laura Del Coco^1^, Francesco Paolo Fanizzi^1^, Alberto Basset^1,2,3^

^1^Department of Biological and Environmental Sciences and Technologies (DiSTeBA), University of Salento, Via Monteroni, I-73100 Lecce, Italy;

^2^National Biodiversity Future Centre (NBFC), Palermo, Italy

^3^LifeWatch ERIC, Service Centre, Lecce, Italy

* Correspondence: federica.decastro@unisalento.it (F.D.C.); ludovico.lezzi@unisalento.it (L.L.)


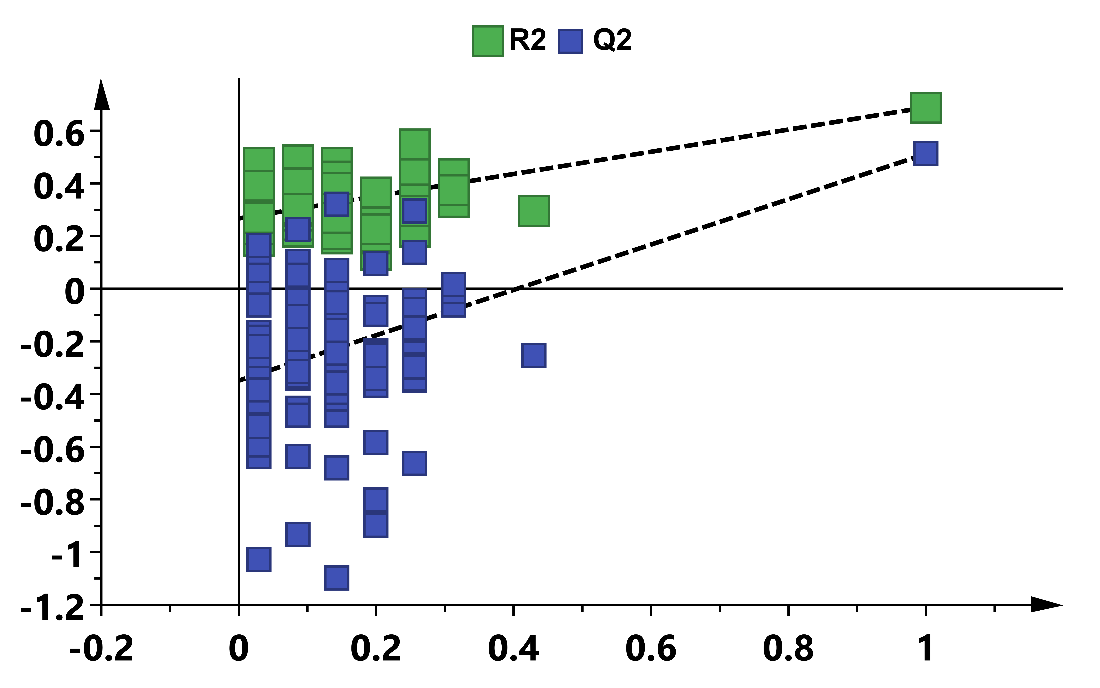


**S1.** Permutation Test (n=100) for the OPLS-DA model of Figure 4.

**Table S1.CV- ANOVA of the OPLS-DA model of Figure 4.**
